# Functional mapping of hospitals by diagnosis-dominant case-mix analysis

**DOI:** 10.1186/1472-6963-7-50

**Published:** 2007-04-10

**Authors:** Kiyohide Fushimi, Hideki Hashimoto, Yuichi Imanaka, Kazuaki Kuwabara, Hiromasa Horiguchi, Kohichi B Ishikawa, Shinya Matsuda

**Affiliations:** 1 Department of Health Care Informatics, Tokyo Medical and Dental University Graduate School, Tokyo, Japan; 2 Department of Health Management and Policy, The University of Tokyo Graduate School of Medicine, Tokyo, Japan; 3 Department of Healthcare Economics and Quality Management, Kyoto University Graduate School of Medicine, Kyoto, Japan; 4 Department of Health Care Administration and Management, Kyushu University Graduate School of Medical Science, Fukuoka, Japan; 5 Cancer Information and Epidemiology Division, National Cancer Center Research Institute, Tokyo, Japan; 6 Department of Preventive Medicine and Community Health, University of Occupational and Environmental Health, Fukuoka, Japan

## Abstract

**Background:**

Principles and methods for the allocation of healthcare resources among healthcare providers have long been health policy research issues in many countries. Healthcare reforms including the development of a new case-mix system, Diagnosis Procedure Combination (DPC), and the introduction of a DPC-based payment system are currently underway in Japan, and a methodology for adequately assessing the functions of healthcare providers is needed to determine healthcare resource allocations.

**Methods:**

By two-dimensional mapping of the rarity and complexity of diagnoses for patients receiving treatment, we were able to quantitatively demonstrate differences in the functions of different healthcare service provider groups.

**Results:**

On average, inpatients had diseases that were 3.6-times rarer than those seen in outpatients, while major teaching hospitals treated inpatients with diseases 3.0-times rarer on average than those seen at small hospitals.

**Conclusion:**

We created and evaluated a new indicator for DPC, the diagnosis-dominant case-mix system developed in Japan, whereby the system was used to assess the functions of healthcare service providers. The results suggest that it is possible to apply the case-mix system to the integrated evaluation of outpatient and inpatient healthcare services and to the appropriate allocation of healthcare resources among health service providers.

## Background

In order to effectively provide a variety of healthcare services, many countries are striving to devise new healthcare policies aimed at assigning appropriate roles, ensuring mutual cooperation among various healthcare service providers, appropriately allocating healthcare resources, and evaluating healthcare service providers [1-4].

For more than 30 years, Japan has maintained a nationwide social health insurance system of the Bismarck type. Almost the entire population of Japan is covered by this system, and it is financed by premiums paid by insured persons and employers, and government compensation. The system in Japan is similar to the German system that was first introduced by Bismarck. On the provider side, the majority of clinics and hospitals are private, though for-profit organisations are not allowed to own or manage hospitals and clinics. The relatively low-cost universal health insurance system of Japan is recognised globally as a major achievement [5]. However, the Japanese system compares unfavourably with those in other developed countries in terms of the inefficiency of healthcare service delivery, as reflected by long hospital stays of more than 20 days as compared to only 6.7 days on average for Organisation for Economic Co-operation and Development (OECD) countries [6]. In addition, the specialist healthcare service provider system has been criticised for being poorly organised with non-systematic delivery of services, as evidenced by the glut of expensive diagnostic equipment such as computed tomography scanners and magnetic resonance imaging units. The number of these devices per 100,000 population is more than five times the average of OECD countries [6].

In Japan, health policy measures have attempted to assign suitable roles and promote cooperation between primary care and specialist healthcare services, based on hospital bed regulations according to regional healthcare programs and incentives via medical service fees [7-9]. However, to date, these attempts have failed to assure the effectiveness of this approach. In this study, we used the Diagnosis Procedure Combination (DPC) case-mix system [10-12] recently developed in Japan to quantitatively analyse differences in outpatient and inpatient healthcare functions according to the type of medical institution. Our aim was to elucidate differences in function among hospitals by using case-mix-based indicators and to examine the possibilities of applying such indicators to health resource allocation in Japan. In evaluating medical institution functions, our study includes the complexity of the patient case mix previously used in such analyses as well as evaluation of the rarity of the case mix, which allows us to assess the provision of healthcare for a wide-range of diseases, including rare conditions for which clinical research or clinical training is vital at teaching hospitals [13,14].

## Methods

We assigned DPC codes for 591 diagnostic categories to the International Statistical Classification of Diseases and Related Health Problems 10th Revision codes for the primary diagnoses of patients in the 1.2 million records of the Patient Survey conducted in 2002 in Japan [15]. DPC is a diagnosis-dominant case-mix system, comprised of 591 higher order disease categories [see Additional file 1] and includes approximately 2,500 lower order categories, such as procedures, co-morbidities and complications. The Patient Survey is conducted every three years and the data for 2002 are the most recently available. In this survey, 70% of hospitals and 7% of clinics were randomly selected for investigation. The number and clinical features of patients discharged from the target facilities during the specified month, and those of patients who visited or stayed at the target hospitals on the specified day are reported. The outpatients are defined as those who did not use hospital beds: those receiving ambulatory surgeries, day treatments, consultations, follow-up visits and radiology tests are counted as outpatients. As primary and advanced cares are not clearly distinguished in Japan and there is virtually no difference between private and public facilities in terms of health services, it is reasonable to assume that all patients are included in this survey and that the data are representative of the entire population of Japan.

We defined the rarity index (Ri) of a DPC diagnostic category as the common logarithm of the relative number of patients [16] who were receiving medical care for a disease in that DPC diagnostic category as follows:

*Ri*_*DPC *_= log_10_(*p*_*DPC*_),

where *Ri*_*DPC *_is the rarity index for a DPC category and *p*_*DPC *_is the relative number of patients who were receiving medical care for a disease in that DPC diagnostic category in Japan. The complexity index (Ci) of a DPC diagnostic category was defined as the relative value of the total admission fee per admission for patients who received inpatient care for a disease in that DPC diagnostic category, which we calculated from the DPC payment schedule [10]. We classified Ri and Ci, calculated for inpatient and outpatient groups for each of the healthcare facilities, as follows: major teaching hospitals (MTH) consisted of 80 university hospitals and two national centre hospitals, other teaching hospitals (OTH) of teaching hospitals other than MTH, other public hospitals (OPH) of non-teaching public hospitals, large private hospitals (LPH) of non-teaching private hospitals with 400 or more beds, small private hospitals (SPH) of non-teaching private hospitals with less than 400 beds, and clinics (C) of healthcare facilities with 0 to 19 beds. The characteristics of the healthcare facility groups in Japan are shown in Table 1. It should be noted that most hospitals in Japan provide considerable outpatient care and that some clinics have small inpatient facilities [7,9]. The averages of Ri and Ci for each healthcare facility group were calculated as follows:

**Table 1:** Characteristics of healthcare facility groups in Japan

	Number of facilities	Average length of hospital stay (Days)	Average number of beds per facility	Total number of outpatients per day (1000 visits)
Major teaching hospital	165	24.1	543	180
Other teaching hospital	337	21.8	507	354
Other public hospital	1,786	24.5	197	599
Large private hospital	683	27.1	230	180
Small private hospital	5,161	28.0	75	573
Clinic	96,305	17.6	2	3,351

RiG=1nG∑nGRiDPC,CiG=1nG∑nGCiDPC,
 MathType@MTEF@5@5@+=feaafiart1ev1aaatCvAUfKttLearuWrP9MDH5MBPbIqV92AaeXatLxBI9gBaebbnrfifHhDYfgasaacH8akY=wiFfYdH8Gipec8Eeeu0xXdbba9frFj0=OqFfea0dXdd9vqai=hGuQ8kuc9pgc9s8qqaq=dirpe0xb9q8qiLsFr0=vr0=vr0dc8meaabaqaciaacaGaaeqabaqabeGadaaakeaafaqabeqacaaabaGaemOuaiLaemyAaK2aaSbaaSqaaiabdEeahbqabaGccqGH9aqpdaWcaaqaaiabigdaXaqaaiabd6gaUnaaBaaaleaacqWGhbWraeqaaaaakmaaqahabaGaemOuaiLaemyAaK2aaSbaaSqaaiabdseaejabdcfaqjabdoeadbqabaaabaaabaGaemOBa42aaSbaaWqaaiabdEeahbqabaaaniabggHiLdGccqGGSaalaeaacqWGdbWqcqWGPbqAdaWgaaWcbaGaem4raCeabeaakiabg2da9maalaaabaGaeGymaedabaGaemOBa42aaSbaaSqaaiabdEeahbqabaaaaOWaaabCaeaacqWGdbWqcqWGPbqAdaWgaaWcbaGaemiraqKaemiuaaLaem4qameabeaaaeaaaeaacqWGUbGBdaWgaaadbaGaem4raCeabeaaa0GaeyyeIuoakiabcYcaSaaaaaa@5525@

where *Ri*_*G *_and *Ci*_*G *_are the average values of Ri and Ci for the facility group G, respectively, *n*_*G *_is the number of patients in the facility group G, and *Ri*_*DPC *_and *Ri*_*DPC *_are Ri and Ci of the DPC category for each patient in the facility group G.

All statistical analyses were performed using Stata 8.0 SE and the level of significance was set at p < 0.05.

## Results

The Ri and Ci of the 591 DPC diagnostic categories ranged from 0.87 to 7.53 and 0.12 to 7.57, respectively. Among the 591 DPC categories, Ci was very weakly correlated with Ri, with a regression coefficient of 0.15 and adjusted R-square of 0.066, indicating very weak correlation between utilization of health care resources for treatment of a disease and the rarity of that disease.

We defined rare diseases as those comprising the first third of patients in the order of rarity, while intermediate diseases accounted for the second third, and common diseases the last one third. We made comparisons among these groups. The DPC disease category names for each rarity group are summarised in Table 2. The most common diseases in Japan were as follows: hypertensive disease without organ damage (Ri = 0.865), type 2 diabetes (excluding diabetic ketoacidosis) and diabetes not elsewhere classified (Ri = 1.326), cataract and other disorders of the lens (Ri = 1.360), metabolic disorders other than diabetes mellitus such as hyperlipidemia (Ri = 1.466), and asthma (Ri = 1.501).

**Table 2:** Case-mix difference by rarity index among healthcare facility groups in Japan

			Disease categories by rarity index		
			
			Rare diseases	Intermediate diseases	Common diseases
Rarity index			2.20–7.53	1.60–2.15	0.87–1.59
DPC disease category name (excerpts)			Pheochromocytoma, paraganglioma; Malignant cardiac tumour; Tuberculous meningitis, meningoencephalitis; Acquired deformation of femur; Orbital tumour	Malignant breast tumour; Tachycardic arrhythmia; Malignant gastric tumour; Chronic sinusitis; Rheumatoid arthritis of upper limb (from shoulder to hand)	Hypertensive disease (without organ damage); Type 2 diabetes (excluding diabetic ketoacidosis) and diabetes not elsewhere classified*; Cataract and other disorders of the lens; Metabolic disorders other than diabetes mellitus; Asthma
Ratio of patients with each disease category for each group of facilities‡ (%)	Inpatient†	MTH	79.9 (79.5 – 80.3)	15.2 (14.9 – 15.6)	4.8 (4.6 – 5.1)
		OTH	74.4 (74.1 – 74.7)	19.7 (19.4 – 20.0)	5.9 (5.7 – 6.1)
		OPH	68.1 (67.8 – 68.4)	26.4 (26.2 – 26.6)	5.5 (5.4 – 5.6)
		LPH	55.5 (55.1 – 55.9)	39.8 (39.4 – 40.2)	4.7 (4.6 – 4.9)
		SPH	54.1 (53.8 – 54.3)	40.6 (40.3 – 40.8)	5.4 (5.2 – 5.5)
		C	49.2 (48.0 – 50.4)	39.3 (38.1 – 40.4)	11.5 (10.8 – 12.3)
	Outpatient†	MTH	58.0 (57.6 – 58.4)	29.9 (29.5 – 30.2)	12.2 (11.9 – 12.4)
		OTH	52.2 (51.9 – 52.5)	31.9 (31.7 – 32.2)	15.8 (15.6 – 16.1)
		OPH	44.9 (44.6 – 45.1)	34.7 (34.5 – 35.0)	20.4 (20.2 – 20.6)
		LPH	40.4 (39.9 – 41.0)	38.7 (38.2 – 39.3)	20.8 (20.4 – 21.3)
		SPH	36.9 (36.5 – 37.3)	39.0 (38.6 – 39.3)	24.1 (23.8 – 24.4)
		C	28.6 (28.4 – 28.8)	39.6 (39.4 – 39.8)	31.8 (31.6 – 32.0)

DPC: Diagnosis-procedure combination, MTH: Major teaching hospital, OTH: Other teaching hospital, OPH: Other public hospital, LPH: Large private hospital, SPH: Small private hospital, C: Clinic

*DPC100070 for Type 2 Diabetes Mellitus (DM) and DPC100340 for Other DM were analysed as one category in this study.

† Pearson's chi-squared test: p < 0.001 both for inpatients and outpatients

‡ Mean (Confidence interval)

Then, case-mix differences in terms of rarity among the hospital groups in Japan were examined (Table 2). The ratios of patients with each category of diseases differed significantly among the hospital groups (p < 0.001). Rare diseases were more frequently observed with outpatient than with inpatient care. Among the hospital groups, rare diseases were prominent in MTH and OTH whereas common diseases were prominent in C, for both inpatient and outpatient care. Our results suggest that the case-mix differs markedly among hospital groups, suggesting substantial differences in functionality. In addition, it was shown that teaching hospitals provide health care services to patients with rarer diseases than other hospitals in both the inpatient and the outpatient setting.

**Figure 1:**
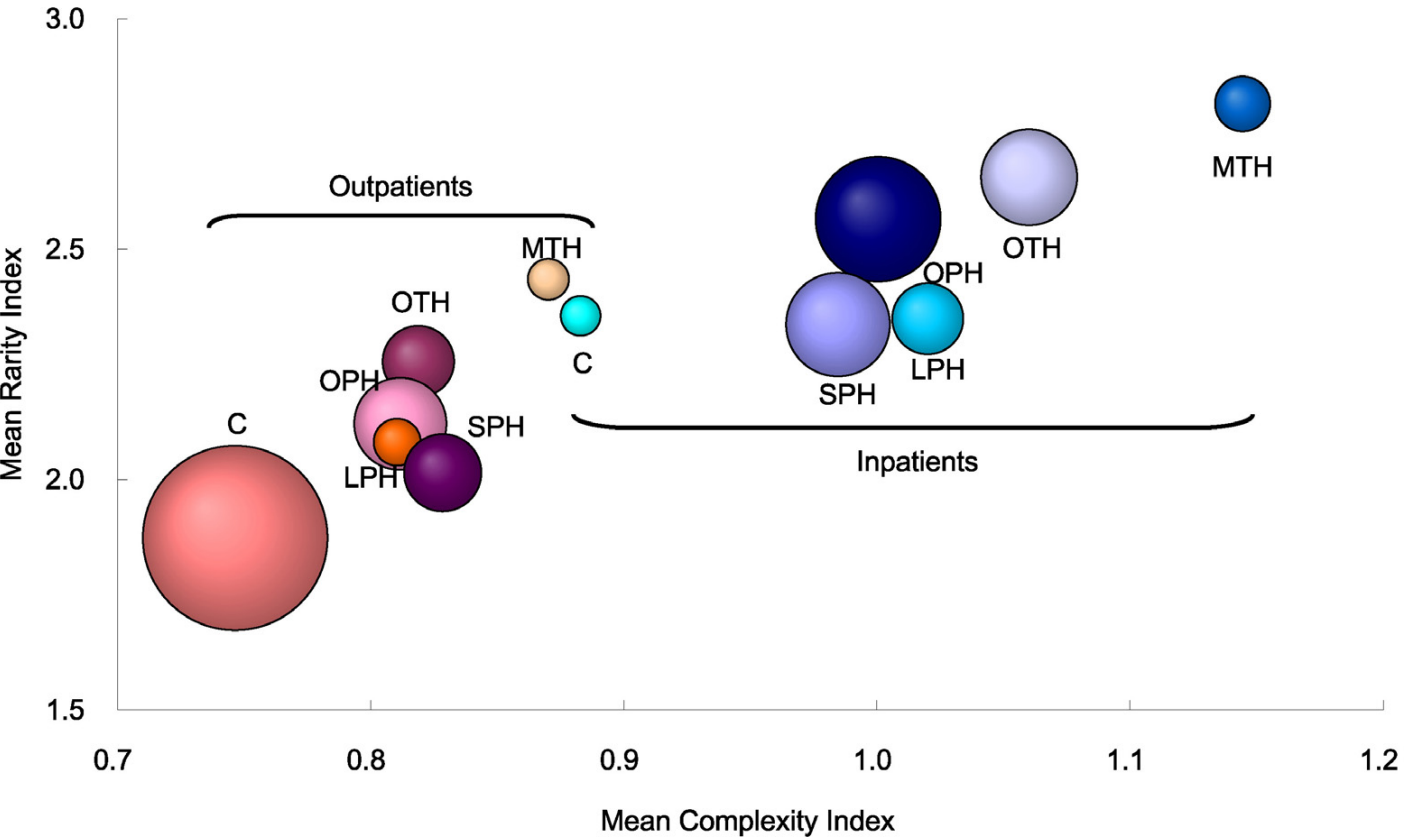
**Rarity and complexity indices for health care providers in Japan**. Mean rarity index, mean complexity index, and relative healthcare expenditures for patients who received ambulatory care and inpatient care at major teaching hospitals (MTH), other teaching hospitals (OTH), other public hospitals (OPH), large private hospitals (LPH), small private hospitals (SPH) and clinics (C). The mean rarity index and mean complexity index per institution are shown. The size of the circle represents the relative healthcare expenditure as determined from the Survey of National Medical Care Insurance Services at 2002.

The averages for the Ri and Ci of inpatient and outpatient care for each healthcare facility group in Japan are summarised in Table 3 and Figure 1. The average values for Ri and Ci were 1.92 and 0.80 among all outpatients, and 2.48 and 1.00 among all inpatients, respectively, indicating that patients with more complex and rarer diseases tended to be hospitalised and that hospitalised patients had diseases that were 3.6 (= 10^0.56^)-times less frequently observed on average than those of outpatients.

**Table 3:** Differences in rarity and complexity among hospital groups in Japan

		Healthcare facility groups					
		
		MTH	OTH	OPH	LPH	SPH	C
In-patient	Rarity index*	2.82 (2.75 – 2.88)	2.66 (2.59 – 2.72)	2.57 (2.50 – 2.63)	2.35 (2.28 – 2.42)	2.34 (2.27 – 2.40)	2.36 (2.26 – 2.45)
		--	p = 0.026 vs. MTH	p < 0.001 vs. MTH	p < 0.001 vs. MTH	p < 0.001 vs. MTH	p < 0.001 vs. MTH
		--	--	p = 0.049 vs. OTH	p < 0.001 vs. OTH	p < 0.001 vs. OTH	p < 0.001 vs. OTH
		--	--	--	p < 0.001 vs. OPH	p < 0.001 vs. OPH	p = 0.001 vs. OPH
		--	--	--	--	N.S. vs. LPH	N.S. vs. LPH
		--	--	--	--	--	N.S. vs. SPH
	Complexity index*	1.14 (1.09 – 1.20)	1.06 (1.01 – 1.11)	1.00 (0.96 – 1.05)	1.02 (0.97 – 1.07)	0.98 (0.94 – 1.03)	0.88 (0.83 – 0.93)
		--	N.S. vs. MTH	p = 0.002 vs. MTH	p = 0.013 vs. MTH	p < 0.001 vs. MTH	p < 0.001 vs. MTH
		--	--	N.S. vs. OTH	N.S. vs. OTH	p = 0.015 vs. OTH	p < 0.001 vs. OTH
		--	--	--	N.S. vs. OPH	N.S. vs. OPH	p = 0.010 vs. OPH
		--	--	--	--	N.S. vs. LPH	p = 0.005 vs. LPH
		--	--	--	--	--	p = 0.023 vs. SPH
	Relative healthcare expenditure†	0.03	0.09	0.15	0.05	0.10	0.02
Out-patient	Rarity index*	2.43 (2.27 – 2.60)	2.26 (2.17 – 2.34)	2.12 (2.05 – 2.19)	2.08 (1.95 – 2.21)	2.02 (1.94 – 2.09)	1.87 (1.84 – 1.91)
		--	N.S. vs. MTH	p < 0.001 vs. MTH	p = 0.001 vs. MTH	p < 0.001 vs. MTH	p < 0.001 vs. MTH
		--	--	p = 0.021 vs. OTH	p = 0.033 vs. OTH	p < 0.001 vs. OTH	p < 0.001 vs. OTH
		--	--	--	N.S. vs. OPH	p = 0.048 vs. OPH	p < 0.001 vs. OPH
		--	--	--	--	N.S. vs. LPH	p = 0.003 vs. LPH
		--	--	--	--		p < 0.001 vs. SPH
	Complexity index*	0.87 (0.77 – 0.97)	0.82 (0.76 – 0.87)	0.81 (0.77 – 0.86)	0.81 (0.73 – 0.89)	0.83 (0.78 – 0.88)	0.75 (0.73 – 0.77)
		--	N.S. vs. MTH	N.S. vs. MTH	N.S. vs. MTH	N.S. vs. MTH	p = 0.020 vs. MTH
		--	--	N.S. vs. OTH	N.S. vs. OTH	N.S. vs. OPH	p = 0.016 vs. OTH
		--	--	--	N.S. vs. OTH	N.S. vs. LPH	p = 0.011 vs. OPH
		--	--	--	--	N.S. vs. LPH	N.S. vs. OPH
		--	--	--	--	--	p = 0.002 vs. SPH
	Relative healthcare expenditure†	0.02	0.05	0.08	0.02	0.06	0.33

MTH: Major teaching hospital, OTH: Other teaching hospital, OPH: Other public hospital, LPH: Large private hospital, SPH: Small private hospital, C: Clinic, N.S.: Not significant

*Mean (confidence interval) and p-values by Student's t test

† Determined from results of the Survey of National Medical Care Insurance Services conducted in 2002 in Japan.

The teaching hospitals were shown to provide health care services to inpatients with relatively rare diseases. The diseases of patients admitted to MTH were 3.0 (= 10^0.479^)-times rarer on average than those of patients admitted to SPH. The averages of the Ri differed significantly among MTH, OTH, OPH and LPH, whereas those for LPH, SPH and C were rather similar. Our results may suggest that there are four functionally different groups, i.e. private and public facilities and two types of teaching hospitals, in terms of the rarity of diseases in the inpatient care setting in Japan. Our results also showed that teaching hospitals provide more complex services than other facilities. However, the differences in Ci among the facility groups were not as distinct as those for Ri, indicating that the Ri may more clearly illustrate differences in hospital function than the Ci.

We also recognised a significant difference in the rarity of outpatient care among the facility groups. There was a 3.6 (= 10^0.561^)-fold difference in the Ri of diseases between MTH and C. On the contrary, in view of complexity, only C had a significantly different profile. Our results clearly demonstrate outpatients visiting clinics to have less complex and more frequently observed diseases than the outpatients visiting hospitals, indicating that outpatient function differs markedly between clinics and hospitals.

Figure 1 suggests a positive correlation between Ri and Ci among the facility groups. However, when examined together with Table 2, it becomes apparent that there are several differences between Ri and Ci in describing the functions of the six facility groups. For inpatient care, Ri appears to have greater power to discriminate functional differences among the facility groups because most of the differences between each pair of groups were statistically significant. We emphasise especially the Ri difference between the public and private hospital groups in contrast to the Ci similarity of these groups. For outpatient care, although Ci was similar in all but the clinics, Ri differed significantly among the facility groups. The results suggest that Ri is superior to Ci in describing the functional differences in outpatient care. In terms of health resource allocation, public and private sectors with intermediate complexity use the majority of resources whereas teaching sectors with high rarity and complexity use less for inpatient care. Clinics consume nearly 60 % of outpatient resources and resource amounts for the hospital groups were lower than for clinics.

## Discussion

The analysis of complexity and rarity based on our case-mix system clearly demonstrated differences in function between Japanese healthcare service provider groups. Regulations governing hospitals in Japan are quite lax, apart from those on bed numbers, and there are almost no policy regulations on the provision of specialised healthcare [7-9]. However, two-dimensional mapping based on complexity and rarity revealed the natural progression in functional specialisation for both outpatient and inpatient care, with various forms of specialised care being provided by clinics, non-teaching hospitals, and university hospitals.

Application of the case-mix system is a new approach to allocating healthcare resources. The DPC, as developed in Japan, provides a diagnosis-dominant disease classification system. DPC has a hierarchical classification system with diagnosis at the top level, procedures at the second, and co-morbidities and complications at the third. When DPC is used to determine the price for inpatient care, cases are classified using all three levels of the DPC classification, which results in approximately 2500 groupings. We assume that when DPC is applied to global resource allocation, since a simpler classification is required, only the diagnostic groupings at the top level can be used. This allows some unique applications which enhance our understanding of regional disease structures [11]. Furthermore, if some reduction in accuracy is feasible, outpatient and inpatient healthcare can be evaluated on a uniform scale.

Moreover, the DPC system allows novel analyses from the perspective of rarity as seen in this study. The evaluation of case-mix rarity may also be applicable to decision-making on the allocation of healthcare resources based on education and training, or advanced healthcare functions. It has been shown that most teaching and public hospitals, although they spend relatively more health care resources per patient than other facilities, tend to have a financing deficit in contrast to other hospitals and clinics in Japan. We consider inadequate resource allocation to probably be due to most health care organisations being financed according to basically the same fee-for-service payment schedule in Japan. In this scheme, as the incomes of hospitals are determined almost entirely by the quantity of health care services they provide, differences in case-mix rarity or complexity among these hospitals may not be reflected in their financing. Teaching hospitals are assumed to need more resources because they are required to provide teaching and research services in addition to clinical care. We speculate that the high Ri obtained herein for teaching hospitals may be related to these additional functions of teaching hospitals. Therefore, we anticipate that our case-mix-based approach will improve assessment of the needs for health care resources in teaching and public hospitals.

A comparison of inpatients of intermediate-level complexity at public and private medical institutions showed an equivalent patient complexity but the rarity of diseases of patients receiving treatment was higher at public medical institutions. This may suggest that the diagnosis and treatment of rare diseases requires higher level training as well more sophisticated diagnostic and other types of equipment, such that public medical institutions, where more funding is received through subsidies, are responsible for providing healthcare services for rare diseases. Among many studies regarding the effects of hospital ownership on quality, cost and clinical behaviours [17-19], our findings may be related to the previous observation of mutual functional compensations between public and private sectors [20]. We speculate that the case-mix differences in private and public sectors reflect the roles and the functions of those sectors. Most intractable diseases, including some congenital diseases, amyloidosis, severe collagen diseases and infectious diseases, are rare, and their diagnosis and treatment are anticipated to be more difficult and health-resource consuming than those for other diseases. Consequently, healthcare for patients with such diseases tends to be provided by public sectors or teaching hospitals in Japan.

As for outpatient care, our results showed that outpatient clinics, which consume approximately one third of total healthcare expenditures, have the lowest Ri and Ci. Japan spends a higher proportion of its total healthcare budget on outpatient healthcare than other developed countries [5]. Although a shift of healthcare resources from inpatient to outpatient services may be one of the measures for improving the efficiency of healthcare systems, we consider the problem in Japan to be whether high outpatient costs counterbalance the quality of services in the outpatient sector. Our results may indicate that care for outpatients with complex and rare diseases is provided in the hospital rather than the clinic sector. Our case-mix analysis thus highlights the potential for healthcare policy research on the reallocation of healthcare resources, for example, by investigating the introduction of a capitation method in place of the current fee-for-service system. We speculate that a capitation-based payment system would be more suitable than the fee-for-service payment system, which is currently applied in Japan, as the incentives for efficient delivery of health care services are very poor with the latter system. We anticipate that investigations on the suitability of a capitation system, using our case-mix-based approach, might show such a payment system to be beneficial.

Many countries have adopted case-mix systems for hospital financing [21]. Health Care Financing Administration-Diagnosis Related Groups (HCFA-DRG) or its derivatives have been used for developing a payment system in the USA as well as for adjustment of hospital budgeting in some countries including Belgium, France and Ireland. Our DPC differs from DRG systems in that DPC uses disease-oriented classification at its primary grouping level. When DPC is used for determining inpatient payments, groupings by procedures, co-morbidities and complications defined at the lower levels are utilised. The primary diagnosis-based level of DPC can be applied to describing the disease structure or case-mix profiling, such as the rarity of a disease, as shown in our study. Limitations of our approach may include its dependence on the rationality of case-mix classification logics.

Also, in terms of rarity assessment, the case-mix-based approach relies on the adequacy of the granularity of the grouping. We consider the grouping logics of DPC to be acceptable, because DPC was designed and has been maintained with consensus among specialist physician boards in Japan and it has in fact been applied to the payment system in acute care hospitals. In addition, we anticipate that the advantages of our method will overcome its limitations, although the use of a diagnosis domain without a procedure domain of DPC logics may lead to ambiguity in grouping logics.

## Conclusion

We have provided one example of a method, using a case-mix system, designed to clarify the differences among healthcare service provider functions based on differences in disease profiles among patients receiving treatment. This method allows a comprehensive evaluation of healthcare service provider functions, including inpatient and outpatient status, as well as teaching and research functions. It could therefore serve as a useful indicator for the appropriate allocation of healthcare resources among different healthcare service providers. It may be applicable to any financial measures including adjustments of hospital budgets, supplemental payments and subsidies.

## List of abbreviations

DPC, diagnosis-procedure combination; Ri, rarity index; Ci, complexity index; MTH, major teaching hospitals; OTH, other teaching hospitals; OPH, other public hospitals; LPH, large private hospitals; SPH, small private hospitals; C, clinics; OECD, Organisation for Economic Co-operation and Development; HCFA-DRG, Health Care Financing Administration-Diagnosis Related Groups

## Competing interests

The author(s) declare that they have no competing interests.

## Authors' contributions

KF performed the quantitative analysis and drafted the manuscript, which was critically reviewed by all authors. SM and HH planned the study, and analysed and interpreted the data. YI, KK, HH, KBI, and SS analysed and interpreted the data. All authors read and approved the final manuscript.

## Pre-publication history

The pre-publication history for this paper can be accessed here:



## Supplementary Material

Additional file 1 **DPC Disease Category Definition Table**. A PDF format file containing a table showing the DPC Category Code, DPC Disease Category Name, and ICD-10 codes classified according to each DPC category. Click here for file
