# Supplementary material for: Functional mapping of hospitals by diagnosis-dominant case-mix analysis
Source: BMC Health Serv Res. 2007 Apr 10;7:50. doi: 10.1186/1472-6963-7-50 (PMC1854890; doi:10.1186/1472-6963-7-50)
Supplement: Additional file 1 — DPC Disease Category Definition Table. A PDF format file containing a table showing the DPC Category Code, DPC Disease Category Name, and ICD-10 codes classified according to each DPC category. [file 1472-6963-7-50-S1.pdf]

DPC Disease Category Definition Table

DPC Category Code	DPC Disease Category Name	International Statistical Classification of Diseases 10 (ICD-10)
010010	Brain tumour	C70*,C71*,C722,C723,C724,C793,D320,D329,D330,D331,D332,D333,D337,D339,D42*,D43*,G131,G132
010020	Subarachnoid haemorrhage, ruptured cerebral aneurysm	I60*
010030	Unruptured cerebral aneurysm	I670,I671
010040	Nontraumatic intracranial haematoma (excluding nontraumatic subdural haematoma)	I61*,I629,I680,Q280,Q281,Q282,Q283
010050	Nontraumatic subdural haemorrhage	I620,I621
010060	Cerebral infarction	G45*,G46*,I63*,I65*,I66*,I675,I679,I693,I978
010070	Cerebrovascular disorder (NEC)	I681,I682,I688,I690,I691,I692,I694,I698
010080	Inflammation with cerebrospinal infection	A858,G00*,G02*,G03*,G041,G042,G048,G049,G05*,G06*,G07,G08,G09,G958
010083	Tuberculous meningitis, meningoencephalitis	A17*,G01
010086	Prion disease	A81*
010090	Multiple sclerosis	G35,G360
010100	Demyelinating disease (NEC)	G040,G361,G368,G369,G37*
010110	Immune-mediated/inflammatory neuropathy	G61*
010111	Hereditary neuropathy	G130,G53*,G58*,G59*,G60*,G62*,G63*,G64
010120	Idiopathic (mono) neuropathy	G50*,G52*
010130	Myasthenia gravis, neuropathy (NEC)	G70*,G731
010140	Muscle disease (NEC)	G71*,G72*,G730,G732,G733,G734,G735,G736,G737
010150	Cerebrovascular disease (NEC)	I64,I672,I676,I677,I678
010155	Spinal muscular atrophy and related syndromes	G12*
010160	Parkinson disease	G20,G21*,G22

010170	Degenerative disease of basal ganglia and other areas	G10,G23*,G254,G255,G903
010180	Involuntary movement	G243,G245,G250,G251,G252,G253,G256,G26
010190	Hereditary ataxia	G11*
010200	Hydrocephalus	G91*
010210	Alzheimer disease	G30*
010220	Dementia (excluding Alzheimer disease)	F01*,G138,G310,G311,G318,G319,G320,I673,I674
010230	Epilepsy	G40*,G41*
010240	Migraine, headache syndrome (NEC)	G43*,G44*
010250	Alcohol dependence syndrome	F102,F107
010260	Wernicke encephalopathy	E512
010270	Toxic encephalopathy	A051,G92
010280	Dystonia, adynamia	G240,G241,G242,G244,G248,G249
010290	Autonomic nervous system disorder	G900,G901,G902,G908,G909
010300	Dyssomnia	G470,G471,G472,G474,G478,G479
010310	Brain disorder (NEC)	G930,G931,G932,G933,G935,G936,G937,G938,G939,G94*,G96*,G97*,G98,G99*
010320	Infectious disease of central nervous system	A82*,A88*,A89,B010,B011,B020,B021,B050,B051,B060
010370	Other nervous system diseases	G258,G259,G312,G328
020010	Malignant tumour of eyelid/ conjunctiva	C441,C690
020020	Malignant orbital tumour	C696
020030	Malignant uveal melanoma	C693,C694
020040	Retinoblastoma	C692
020050	Malignant ocular tumour	C691,C698,C699
020060	Benign ocular tumour	D031,D221,D231,D319
020070	Conjunctival/corneal tumour	D310,D311
020080	Orbital tumour	D316
020090	Tumour of iris/ciliary body/choroid/ retina	D312,D313,D314
020100	Lachrymal sac tumour	C695,D315
020110	Cataract and other disorders of lens	H25*,H260,H262,H263,H264,H268,H269,H27*,H28*,Q12*

020120	Acute anterior uveitis	H200,H209
020130	Harada disease	H308
020140	Chorioretinitis/retinitis/acute retinal necrosis	H300,H301,H302,H309
020150	Strabismus (excluding traumatic/ adhesive strabismus)	H49*,H500,H501,H502,H503,H504,H505,H506, H508,H509
020160	Arhegmatogenous retinal detachment	H332,H334
020170	Rhegmatogenous retinal detachment	H330,H333
020180	Diabetic hyperplastic retinopathy	H360
020190	Retinopathy of prematurity	H351
020200	Macular/posterior pole degeneration	H353,H359
020210	Obstruction of retinal blood vessels	H34*,H350,H352
020220	Glaucoma	H400,H401,H402,H404,H405,H406,H408,H409, H420,H428,H445,Q13*,Q150
020230	Blepharoptosis	H024,Q100
020240	Vitreous body disease	H43*
020250	Conjunctival disorder	H10*,H11*,H13*
020270	Scleral disorder	H15*
020280	Corneal disorder	H16*,H17*,H18*,H19*
020290	Dacryostegnosis/lachrymal insufficiency	H045
020300	Lachrymal canaliculitis, dacryocystitis	H043,H044
020310	Lachrymal fistula	H046
020320	Disease of eyelid, lachrymal system and orbit	H00*,H01*,H020,H021,H022,H023,H025,H026, H027,H028,H029,H03*,H040,H041,H042,H048, H049,H06*
020340	Iridocyclitis, disorder of iris/ciliary body	H201,H202,H208,H21*,H22*
020350	Choroidal disease	H31*,H32*,H331,H354,H355,H356,H357,H358, H368
020360	Eyeball disorder	H440,H441,H442,H443,H448,H449,H45*
020370	Optic nerve disorder	H471,H472,H473,H474,H475,H476,H477,H48*
020380	Ocular motor dysfunction	H51*,H55
020390	Vision/visual field disorder	H52*,H53*,H54*

020400	Disorder of eye and adnexa	H57*,H58*,H59*
020410	Inflammation and disorder of optic nerve	H46,H470,S040
030010	Malignant tumour of oral cavity/lower jaw and lips	C00*,C02*,C03*,C04*,C050,C058,C059,C06*,C14*,C411
030050	Malignant tumour of salivary glands	C07,C08*
030060	Malignant tumour of epipharynx	C11*
030070	Malignant tumour of mesopharynx	C01,C051,C052,C09*,C10*
030080	Malignant tumour of hypopharynx	C12,C13*
030090	Malignant laryngeal tumour	C32*
030100	Malignant tumour of nasal cavity/paranasal sinuses	C300,C31*,C410
030110	Metastatic malignant tumour of neck	C770
030130	Malignant tumour of auditory organs	C301,C442
030140	Malignant tumour of head and neck (NEC)	C430,C431,C432,C433,C434,C440,C443,C444,C470,C490,C760
030150	Tumour of oral cavity/pharynx	D000,D020,D023,D032,D042,D100,D101,D102,D103,D109,D140,D141,D370,I860
030160	Benign tumour of major salivary glands	D11*
030170	Tumour of middle ear, respiratory system and intrathoracic organs	D385
030180	Stomatitis, disorder of oral cavity	K12*,K13*
030190	Sialadenitis, abscess of salivary glands	K112,K113
030200	Intraglandular sialolithiasis	K115
030210	Benign tumour of oral cavity, tonsil and pharynx	D104,D105,D106,D107
030220	Ranula	K116
030230	Chronic disease of tonsils and adenoids	J35*
030240	Peritonsillar abscess	J36
030245	Infectious mononucleosis	B27*
030250	Sleep apnoea	G473
030270	Upper respiratory tract inflammation	J00,J02*,J06*,J31*

030280	Vocal cord polyp, vocal cord nodule	J381,J382
030290	Vocal cord palsy	J380
030300	Vocal cord disease (NEC)	D380,J383,J384,J385,J386,J387
030320	Nasal septal deviation	J342
030330	Acute sinusitis	J01*
030340	Vasomotor rhinitis, allergic rhinitis	J30*
030350	Chronic sinusitis	J32*
030360	Paranasal sinus cyst, nasal vestible cyst	J341,J348
030370	Nasal polyp	J33*
030380	Epistaxis	R040
030390	Facial nerve disorder	G51*
030400	Vestibular dysfunction	H810,H811,H812,H813,H818,H819
030410	Vertigo of central origin	H814
030423	Acute perceptive deafness	H831,H910
030425	Other auditory disorders	H833,H90*,H911,H913,H918,H919,H93*
030428	Sudden idiopathic hearing loss	H912
030430	Nonsuppurative otitis media, eustachian salpingitis and obstruction	H65*,H68*
030440	Chronic suppurative otitis media/ middle ear cholesteatoma	H661,H662,H663,H669,H70*,H71,H950
030450	External ear disorder (NEC)	H60*,H61*,H62*
030460	Disorder of middle ear/mastoid	B053,H660,H664,H67*,H69*,H72*,H73*,H74*, H75*
030470	Inner ear disorder (NEC)	H82,H830,H832,H838,H839
030475	Otosclerosis	H80*,Q163
030480	Ear disorder (NEC)	H94*
030490	Upper respiratory tract disease (NEC)	J340,J343,J37*
030500	Salivary glands disease (NEC)	K110,K111,K114,K117,K118,K119
030520	Disorder of ear/mastoid after treatment	H951,H958,H959
040010	Malignant mediastinal tumour, malignant tumour of mediastinum/ pleura	C37,C381,C382,C383,C388,C39*,C771,C781,C783

040020	Benign tumour of mediastinum	D150,D152,D174,D383,D384,E320,E329,Q341
040030	Benign tumour of respiratory system	D142,D143,D144,D381,D386
040040	Malignant pulmonary tumour	C33,C34*,C780,D021,D022,D024
040050	Chest wall tumour, pleural tumour	C384,C450,C452,C782,D190,D382
040060	Acute tonsillitis, acute laryngopharyngitis	A691,J03*,J04*,J05*,J390,J391,J392,J393,J398,J399
040070	Influenza, viral pneumonia	J10*,J11*,J12*
040080	Pneumonia, acute bronchitis, acute bronchiolitis	A481,B012,B052,B371,B59,J13,J14,J15*,J16*,J17*,J18*,J20*,J21*,J22
040090	Lower respiratory tract infection (NEC)	J40,J41*,J42
040100	Asthma	J45*,J46
040110	Interstitial pneumonia	J60,J61,J62*,J63*,J64,J65,J66*,J67*,J68*,J69*,J70*,J82,J84*,J990,J991
040120	Chronic obstructive lung disease	J43*,J44*
040130	Respiratory failure (NEC)	J96*
040140	Airway haemorrhage (NEC)	R042,R048,R049
040150	Pulmonary/mediastinal infection and abscess	A065,B440,B441,B45*,B659,B664,E321,E328,J850,J851,J852,J853,J86*,J985
040160	Respiratory tuberculosis	A160,A161,A162,A163,A165,A167,A168,A169,A19*
040170	Mycobacterial infection (excluding tuberculosis)	A310,A319
040180	Respiratory airflow disorder including bronchial stenosis	A164,J980,J981
040190	Pleural effusion, pleural disease (NEC)	J90,J91,J92*,J941,J948,J949
040200	Pneumothorax	J93*
040210	Bronchiectasis	J47
040220	Diaphragmatic tumour/diaphragmatic disease congenital malformation of hernia	J986,K44*,Q401,Q790,Q791
040230	Haemothorax, haemopneumothorax, chylothorax	J940,J942,T792
040240	Pulmonary circulation disorder	I271,I28*,J80,J81,T790,T791

040250	Cor pulmonale	I278,I279
040260	Primary pulmonary hypertension	I270
040310	Other respiratory disorders	J95*,J982,J983,J984,J988,J989,J998
050010	Malignant cardiac tumour	C380
050020	Benign cardiac tumour	D151
050030	Acute myocardial infarction, recurrent myocardial infarction	I21*,I22*,I24*
050040	Complication following acute myocardial infarction	I23*
050050	Angina pectoris, chronic ischemic heart disease	I20*,I25*
050060	Cardiomyopathy	I421,I422,I423,I424,I425,I426,I427,I428,I429,I43*
050065	Dilated cardiomyopathy	I420
050070	Tachycardiac arrhythmia	I456,I47*,I48,I490,I491,I492,I493,I494,I498
050080	Valvular disorder	I05*,I06*,I07*,I34*,I35*,I36*,I37*
050085	Combined valvular disease	I08*
050090	Endocarditis	I33*,I38
050100	Myocarditis	I40*,I41*,I514
050110	Acute pericarditis	I010,I018,I30*,I318,I32*
050120	Constrictive endocarditis	I310,I311
050130	Heart failure	I50*
050140	Hypertensive disease (without organ damage)	I10
050150	Hypertensive disease (with organ damage)	I11*,I13*
050161	Dissecting aneurysm	I710
050162	Ruptured aneurysm	I711,I713,I715,I718
050163	Unruptured aneurysm, iliac aneurysm	I712,I714,I716,I719,I723
050170	Occlusive arterial disease	I700,I702,I708,I709,I720,I721,I724,I73*,I740,I741,I742,I743,I744,I745,I748,I749
050180	Phlebitis, varicose veins of lower extremity	I80*,I821,I828,I829,I83*
050190	Pulmonary embolism	I26*,I822

050200	Cardiovascular disease (NEC)	I011,I012,I019,I02*,I09*,I728,I77*,I95*,I970,I971,I979,I980,I981,I988,I99
050210	Bradyarrhythmia	I440,I441,I442,I443,I444,I445,I446,I447,I450,I451,I452,I453,I454,I455,I458,I459,I46*,I495,T821
050340	Other cardiovascular disorders	I00,I312,I313,I319,I39*,I499,I510,I511,I512,I513,I515,I516,I517,I518,I519,I52*,I729,I780,I788,I789,I79*,I846,I868,I870,I871,I872,I878,I879
060010	Malignant tumour of oesophagus (including cervical region)	C150,C151,C152,C153,C154,C155,C158,C159,D001
060020	Malignant gastric tumour	C16*,D002
060030	Malignant imtestinal tumour	C17*,C268,C451,C480,C481,C482,C488,C772,C784,D017
060035	Malignant tumour of colon (ascending to sigmoid colon)	C18*,C260,C269,C775,C785,D010
060040	Malignant tumour of rectum and anus (rectosigmoid colon to anus)	C19,C20,C21*,D011,D012,D013,D014
060050	Malignant tumour of liver/intrahepatic bile duct (including secondary tumour)	C22*,C787,D015,D376
060060	Malignant tumour of gallbladder	C23
060065	Malignant tumour of extrahepatic bile duct	C24*
060070	Pancreatic/splenic tumour	C25*,C261,D136,D137,D377
060080	Benign oesophageal tumour	D130
060090	Benign gastric tumour	D131,D132,D371
060100	Benign disease of small and large intestine (including benign tumour)	D12*,D133,D191,D197,D199,D201,D372,D373,D374,D375,K57*,K620,K621
060110	Benign hepatic tumour	D134
060120	Portal hypertension (including varicosities of oesophagus, stomach and digestive tract)	I81,I850,I859,I864,I982,K766

060130	Inflammation of oesophagus, stomach, duodenum and other intestines (other benign diseases)	A040,A041,A042,A043,A044,A045,A046,A047,A048,A049,B054,I880,K20,K21*,K220,K221,K222,K223,K224,K225,K226,K228,K229,K23*,K27*,K28*,K29*,K30,K310,K313,K315,K316,K318,K52*,K58*,K627,K633,K634,K638,K639,K90*,K910,K911,K912,K914,K92*,K93*
060140	Gastroduodenal ulcer, gastric diverticulum, pyloric stenosis	K25*,K26*,K311,K312,K314
060150	Appendicitis	K35*,K36,K37,K38*
060160	Inguinal hernia	K40*
060170	Ventral herniation without obstruction or gangrene	K41*,K42*,K43*,K45*,K46*
060180	Crohn disease (NEC)	K50*,K626
060185	Ulcerative colitis	K51*,K593
060190	Ischemic enterocolitis	K55*
060200	Intussusception	K561
060210	Intestinal obstruction without hernia	K560,K562,K563,K564,K565,K566,K567,K913
060220	Anal prolapse	K622,K623
060230	Perirectal abscess	K610,K611,K612,K613,K614,K628
060235	Haemorrhoid	K603,K604,K605
060240	External haemorrhoid	I843,I844,I845,I847
060245	Internal haemorrhoid	I840,I841,I842,I848,I849,K625
060250	Condyloma acuminatum	A630
060260	Anal stenosis, anal fissure	K600,K601,K602,K624
060270	Fulminant hepatitis, acute liver failure, acute hepatitis	B150,B160,B162,B19*,K720,K762
060275	Acute hepatitis (including acute exacerbation of chronic liver disease, excluding acute hepatitis C)	B159,B161,B169,B170,B172,B178,K710,K711,K712,K719,K763
060278	Acute hepatitis C	B171
060280	Alcoholic liver disease	K70*
060290	Chronic hepatitis (excluding chronic hepatitis C)	B180,B181,B188,B189,K713,K714,K715,K716,K718,K73*,K753,K758,K760,K761,K77*
060295	Chronic hepatitis C	B182

060300	Cirrhosis (including biliary cirrhosis)	I820,K717,K721,K729,K740,K741,K742,K743,K744,K745,K746,K765
060310	Hepatic abscess (including bacterial/ parasitic disease)	A064,K750,K751,K752
060320	Hepatic cyst	K768
060330	Gallbladder disease (cholecystolithiasis etc.)	K802,K808
060335	Hydrops of gallbladder, cholecystitis (NEC)	D135,K800,K801,K81*,K820,K821,K822,K823,K824,K828,K829,K835,K870
060340	Bile duct (intra/extra hepatic) lithiasis	K805
060345	Cholangitis	K803,K804,K830,K831,K832,K833,K834,K838,K839,K915
060350	Acute pancreatitis	B263,K85,K871
060360	Chronic pancreatitis (including pancreatic cyst)	K860,K861,K862,K863,K868,K869
060370	Peritonitis, abdominal abscess (excluding female genital organs)	A183,K630,K631,K632,K65*,K67*
060560	Other oral cavity disorders	K00*,K01*,K02*,K03*,K04*,K05*,K06*,K08*,K09*,K10*,K14*
060565	Jaw deformity	K07*
060570	Other digestive tract disorders	K319,K629,K66*,K759,K764,K769,K918,K919
060580	Constipation	K590,K591,K592,K594,K598,K599
070010	Benign tumour of bone or soft tissue (excluding spine and spinal cord)	D160,D161,D162,D163,D167,D168,D169,D179,D211,D212,D214,D216,D219,D481,L72*,M850*,M853*,M854*,M855*,M856*
070015	Benign tumour of skin	D170,D171,D172,D173,D210,D225,D226,D227,D235,D236
070020	Benign tumour of nervous system	D237,D361,Q850
070030	Spinal/spinal cord tumour	C412,C414,C720,C721,C725,C728,C729,C763,D166,D321,D334,G550,M495*
070040	Malignant tumour of bone or soft tissue (excluding spine and spinal cord)	C400,C401,C402,C403,C408,C409,C413,C418,C419,C471,C472,C473,C474,C475,C476,C478,C479,C491,C492,C493,C494,C495,C496,C498,C499,C764,C765,C773,C774,C795,C96*,D092,D097

070042	Malignant tumour of skin including upper limb	C446,C447,C448,C449,C792,D041,D044,D045,D046,D047,D048,D049
070045	Melanoma	C435,C436,C437,C438,C439,D030,D033,D034,D035,D036,D037,D038,D039
070050	Arthritis of shoulder, other disorders of shoulder	M0001,M0011,M0021,M0081,M0091,M0101,M0121,M0131,M0141,M0151,M0161,M0181,M0201,M0211,M0221,M0301,M0311,M0321,M0361,M0711,M0712,M0741,M0751,M0761,M1241,M1251,M1281,M1301,M1311,M1312,M1381,M1382,M1391,M1392,M1901,M1911,M1921,M1981,M1991,M2501,M2511,M2512,M2513,M2541,M2571,M2581,M751,M758,M759
070060	Arthritis of hand and elbow	M0002,M0003,M0004,M0012,M0013,M0014,M0022,M0023,M0024,M0082,M0083,M0084,M0092,M0093,M0094,M0102,M0103,M0104,M0112,M0113,M0114,M0122,M0123,M0124,M0132,M0133,M0134,M0142,M0143,M0144,M0152,M0153,M0154,M0162,M0163,M0164,M0182,M0183,M0184,M0202,M0203,M0204,M0212,M0213,M0214,M0222,M0223,M0224,M0302,M0303,M0304,M0313,M0314,M0322,M0323,M0324,M0362,M0363,M0364,M0713,M0714,M1304,M1313,M1314,M1383,M1384,M1393,M1394,M2514,M2515
070070	Osteomyelitis of upper limb	M0111,M8601,M8602,M8603,M8604,M8611,M8612,M8613,M8614,M8621,M8622,M8623,M8624,M8631,M8632,M8633,M8634,M8641,M8642,M8643,M8644,M8651,M8652,M8653,M8654,M8661,M8662,M8663,M8664,M8681,M8682,M8683,M8684,M8691,M8692,M8693,M8694

070080	Inflammation including synovitis, tenosynovitis and chondritis	M0167,M0207,M0217,M0227,M0747,M0757,M0767,M1246,M1247,M1256,M1257,M1286,M1287,M1307,M1317,M1387,M1397,M1907,M1917,M1927,M1987,M1997,M2420,M2421,M2425,M2428,M2429,M2507,M2547,M2577,M2587,M2597,M650*,M651*,M652*,M653*,M654*,M658*,M659*,M68*,M700*,M701*,M702*,M703*,M706*,M707*,M7080,M7081,M7082,M7083,M7084,M7086,M7087,M7088,M7089,M709*,M7100,M7101,M7102,M7103,M7104,M7105,M7106,M7107,M7108,M7109,M7110,M7111,M7112,M7113,M7114,M7115,M7116,M7117,M7118,M7119,M712*,M713*,M714*,M715*,M718*,M719*,M730*,M731*,M738*,M753,M754,M755,M760*,M761*,M762*,M764*,M765*,M766*,M767*,M768*,M769*,M770*,M771*,M772*,M773*,M775*,M778*,M779*,M794*,M940*,M941*,M948*,M949*
070090	Myositis (including infection)	M600*,M601*,M602*,M608*,M609*,M610*,M611*,M612*,M613*,M614*,M615*,M619*,M630,M631,M632,M638,M752,M791*,M792*,M793*,M795*,M7962,M7963,M7964,M7966,M7967
070100	Suppurative/tuberculous coxitis	M0005,M0015,M0025,M0085,M0095,M0105,M0115,M0125,M0135,M0145,M0155,M0165,M0185,M0205,M0215,M0225,M0305,M0315,M0325,M0365,M2516
070110	Osteomyelitis of periphery of hip joint	M8605,M8615,M8625,M8635,M8645,M8655,M8665,M8685,M8695
070120	Suppurative arthritis of knee	M0006,M0016,M0026,M0086,M0096,M0106,M0116,M0126,M0136,M0146,M0156,M0166,M0186,M0306,M0316,M0326,M0366,M1306,M1316,M1386,M1396

070130	Myelitis of lower limb (excluding hip joint)	M0007,M0017,M0027,M0087,M0097,M0107,M0117,M0127,M0137,M0147,M0157,M0187,M0307,M0317,M0327,M0367,M0717,M2517,M8606,M8607,M8616,M8617,M8626,M8627,M8636,M8637,M8646,M8647,M8656,M8657,M8666,M8667,M8686,M8687,M8696,M8697
070140	Cerebral palsy	G80*,G81*,G82*,G830,G831,G832,G833,G838,G839
070150	Neuropathy of upper limb (including thoracic outlet syndrome)	G54*,P140,P141,P143
070160	Peripheral nerve palsy of upper limb	G56*,P148,P149
070170	Neurological disorder of lower limb	G57*
070180	Spinal deformity	M400*,M401*,M402*,M403*,M404*,M405*,M410*,M411*,M412*,M413*,M414*,M415*,M418*,M419*,M438*,M439*,M962,M963,M964,M965,Q675,Q761,Q762,Q763,Q764
070190	Upper limb deformity	M200,M2101,M2102,M2103,M2104,M2111,M2112,M2113,M2114,M2121,M2122,M2123,M2124,M2132,M2133,M2134,M2152,M2153,M2154,M2171,M2172,M2173,M2174,M2181,M2182,M2183,M2184,M2191,M2192,M2193,M2194
070200	Arthropathy of hand (including degenerative disease)	M0742,M0743,M0744,M0752,M0753,M0754,M0762,M0763,M0764,M1242,M1243,M1244,M1252,M1253,M1254,M1282,M1283,M1284,M151,M152,M18*,M1902,M1903,M1904,M1905,M1912,M1913,M1914,M1915,M1922,M1923,M1924,M1925,M1982,M1983,M1984,M1985,M1992,M1993,M1994,M1995,M2502,M2503,M2504,M2542,M2543,M2544,M2553,M2563,M2572,M2573,M2574,M2582,M2583,M2584,M2592,M2593,M2594
070210	Lower limb deformity	M201,M202,M203,M204,M205,M206,M2105,M2106,M2107,M2115,M2116,M2117,M2125,M2126,M2127,M2136,M2137,M214*,M2156,M2157,M216*,M2176,M2177,M2186,M2187,M2196,M2197,M2506,M670

070220	Arthropathy of hip (including degenerative disease)	M0745,M0755,M0765,M1245,M1255,M1285,M1315,M1385,M1395,M16*,M1906,M1916,M1926,M1986,M1996,M2175,M2185,M2195,M2505,M2545,M2575,M2585,M2595
070230	Arthropathy of knee (including degenerative disease)	M146,M17*,M2546,M2576,M2586,M2596
070240	Flail joint disease (shoulder, hand, leg, knee etc.)	M2422,M2423,M2424,M2426,M2427,M2432,M2433,M2434,M2436,M2437,M2442,M2443,M2444,M2446,M2447,M2520,M2521,M2522,M2523,M2524,M2525,M2526,M2527,M2528,M2529,M2530,M2531,M2532,M2533,M2534,M2536,M2537,M2538,M2539
070250	Internal derangement intra-articular loose body (hip, shoulder, hand, elbow, foot)	M240*,M241*,M2482,M2483,M2484,M2485,M2486,M2487,M2490,M2492,M2493,M2494,M2495,M2496,M2497,M2498,M2499
070270	Knee joint instability	M22*
070280	Osteochondrosis, osteochondral disorder/osteonecrosis, disorder of knee joint during development	M420*,M421*,M429*,M774*,M8700,M8701,M8702,M8703,M8704,M8706,M8707,M8708,M8709,M8710,M8711,M8712,M8713,M8714,M8716,M8717,M8718,M8719,M8720,M8721,M8722,M8723,M8724,M8726,M8727,M8728,M8729,M8730,M8731,M8732,M8733,M8734,M8736,M8737,M8738,M8739,M8780,M8781,M8782,M8783,M8784,M8786,M8787,M8788,M8789,M8790,M8791,M8792,M8793,M8794,M8796,M8797,M8798,M8799,M8808,M8880,M8881,M8882,M8883,M8884,M8885,M8886,M8887,M8888,M8889,M896*,M910*,M912*,M918*,M919*,M920,M921,M922,M923,M924,M925,M926,M927,M928,M931,M939,M942*,M943*
070290	Articular contracture of shoulder	M2451,M2461,M2481,M2491,M2561,M2591,M6241,M750
070300	Contracture/rigidity of cubital/hand joints	M2452,M2453,M2454,M2462,M2463,M2464,M2562,M2564,M6244
070310	Contracture of hip joint	M2455,M2465,M247*,M2565,M6245

070320	Contracture of knee/hip joint	M2456,M2457,M2466,M2467,M2566,M2567,M6246
070330	Spinal inflammation (including infection)	M1308,M1318,M1388,M1398,M460*,M461*,M462*,M463*,M464*,M465*,M468*,M469*,M490*,M491*,M492*,M493*
070340	Spondylosis and other dorsopathies	G551,G552,G553,G558,G950,G951,G952,G959,M432*,M470*,M471*,M472*,M478*,M479*,M480*,M481*,M482*,M483*,M484*,M485*,M488*,M489*,M494*,M498*,M530*,M531*,M533*,M538*,M539*,M995*,M996*,M997*
070350	Intervertebral disk degeneration, disk herniation	M50*,M51*
070360	Unstable vertebra	M430*,M431*,M433*,M434*,M435*,M532*
070370	Spinal osteoporosis	M800*,M801*,M802*,M803*,M804*,M805*,M808*,M809*,M810*,M811*,M812*,M813*,M814*,M815*,M816*,M818*,M819*,M820*,M821*,M828*,M851*
070380	Ganglion	M672,M673,M674
070390	Fibroblastic disorder	M720*,M721*,M722*,M723*,M724*,M728*,M729*
070395	Necrotizing fasciitis	A480,A483,M725*
070400	Avascular necrosis of femoral head	M8705,M8715,M8725,M8735,M8785,M8795
070410	Trigger hip	M2535,M7085,M763*
070420	Slipped capital femoral epiphysis	M930
070430	Neural dystrophia, developmental disorder of bone, osteopathy (NEC)	M890*,M891*,M892*,M893*,M894*,M895*,M898*,M899*
070440	Pigmented villonodular synovitis	M1220,M1221,M1222,M1223,M1224,M1225,M1226,M1227,M1228,M1229
070450	Acquired deformation of femur	M958
070460	Perthes disease of hip joints	M911*
070470	Rheumatoid arthritis of spine, ankylosing spondylitis	M0721,M0722,M0723,M0724,M0725,M0727,M0728,M0729,M45*

070480	Rheumatoid arthritis of upper limb (from shoulder to hand)	M0231,M0232,M0233,M0234,M0281,M0282,M0283,M0284,M0291,M0292,M0293,M0294,M0501,M0502,M0503,M0504,M0511,M0512,M0513,M0514,M0521,M0522,M0523,M0524,M0531,M0532,M0533,M0534,M0581,M0582,M0583,M0584,M0591,M0592,M0593,M0594,M0601,M0602,M0603,M0604,M0611,M0612,M0613,M0614,M0621,M0622,M0623,M0624,M0631,M0632,M0633,M0634,M0641,M0642,M0643,M0644,M0681,M0682,M0683,M0684,M0691,M0692,M0693,M0694,M0701,M0702,M0703,M0704,M0731,M0732,M0733,M0734,M0801,M0802,M0803,M0804,M0821,M0822,M0823,M0824,M0831,M0832,M0833,M0834,M0841,M0842,M0843,M0844,M0881,M0882,M0883,M0884,M0891,M0892,M0893,M0894,M1231,M1232,M1233,M1234
070490	Rheumatoid arthritis of lower limb (excluding hip joint)	M0206,M0216,M0226,M0236,M0237,M0286,M0287,M0296,M0297,M0506,M0507,M0516,M0517,M0526,M0527,M0536,M0537,M0586,M0587,M0596,M0597,M0606,M0607,M0616,M0617,M0626,M0627,M0636,M0637,M0646,M0647,M0686,M0687,M0696,M0697,M0706,M0707,M0716,M0726,M0736,M0737,M0746,M0756,M0766,M0806,M0807,M0826,M0827,M0836,M0837,M0846,M0847,M0886,M0887,M0896,M0897,M1236,M1237
070500	Rheumatoid arthritis of hip joint	M0235,M0285,M0295,M0505,M0515,M0525,M0535,M0585,M0595,M0605,M0615,M0625,M0635,M0645,M0685,M0695,M0705,M0735,M0805,M0825,M0835,M0845,M0885,M0895,M1235
070510	Gout, other joint disorders (NEC)	M10*,M11*,M140*,M141*,M143*,M144*,M145*,M148*,M790*
070520	Disease of lymph node/ lymphatics	A182,D360,I881,I888,I889,I89*,L04*
070530	Radiation-induced skin disorder	L57*,L58*,L598,L599
070540	Clavus	L84,L852,L858,L859,L86
070550	Decubitus ulcer	L89,M0715

070560	Autoimmune disease with systemic organ disease	D86*,L405,L88,L92*,L93*,L940,L941,L942,L943,L95*,L982,L983,M0500,M0510,M0520,M0530,M0580,M0590,M0600,M0610,M0620,M0630,M0640,M0680,M0690,M0710,M0720,M0730,M0740,M0750,M0760,M0800,M081*,M0820,M0830,M0840,M0880,M0890,M09*,M1310,M1380,M1390,M150,M153,M154,M158,M159,M1900,M300,M301,M302,M308,M31*,M32*,M330,M331,M332,M339,M34*,M350,M351,M352,M353,M354,M355,M356,M357,M358,M359,M36*,M633
070570	Scar contracture	L905,L91*,L984,L988
070580	Torticollis	M436*,Q680
071030	Other musculoskeletal/connective tissue diseases	M!!!!,M0000,M0008,M0009,M0010,M0018,M0019,M0020,M0028,M0029,M0080,M0088,M0089,M0090,M0098,M0099,M0100,M0108,M0109,M0110,M0118,M0119,M0120,M0128,M0129,M0130,M0138,M0139,M0140,M0148,M0149,M0150,M0158,M0159,M0160,M0168,M0169,M0180,M0188,M0189,M0200,M0208,M0209,M0210,M0218,M0219,M0220,M0228,M0229,M0230,M0238,M0239,M0280,M0288,M0289,M0290,M0298,M0299,M0300,M0308,M0309,M0310,M0312,M0318,M0319,M0320,M0328,M0329,M0360,M0368,M0369,M0508,M0509,M0518,M0519,M0528,M0529,M0538,M0539,M0588,M0589,M0598,M0599,M0608,M0609,M0618,M0619,M0628,M0629,M0638,M0639,M0648,M0649,M0688,M0689,M0698,M0699,M0700,M0708,M0709,M0718,M0719,M0738,M0739,M0748,M0749,M0758,M0759,M0768,M0769,M0808,M0809,M0828,M0829,M0838,M0839,M0848,M0849,M0888,M0889,M0898,M0899,M120*,M121*,M1230,M1238,M1239,M1240,M1248,M1249,M1250,M1258,M1259,M1280,M1288,M1289,M1300,M1302,M1303,M1305,M1309,M1319,M1389,M1399,M1908,M1909,M1910,M1918,M1919,M1920,M1928,M1929,M198

		0,M1988,M1989,M1990,M1998,M1999,M2100,M2108,M2109,M2110,M2118,M2119,M2120,M2128,M2129,M2170,M2178,M2179,M2180,M2188,M2189,M2190,M2198,M2199,M2450,M2458,M2459,M2460,M2468,M2469,M2480,M2488,M2489,M2500,M2508,M2509,M2510,M2518,M2519,M2540,M2548,M2549,M2550,M2551,M2552,M2554,M2555,M2556,M2557,M2558,M2559,M2560,M2568,M2569,M2570,M2578,M2579,M2580,M2588,M2589,M2590,M2598,M2599,M540*,M541*,M542*,M543*,M544*,M545*,M546*,M548*,M549*,M671,M678,M679,M798*,M799*,M8300,M8301,M8302,M8303,M8304,M8305,M8307,M8308,M8309,M8310,M8311,M8312,M8313,M8314,M8315,M8317,M8318,M8319,M8320,M8321,M8322,M8323,M8324,M8325,M8327,M8328,M8329,M8330,M8331,M8332,M8333,M8334,M8335,M8337,M8338,M8339,M8340,M8341,M8342,M8343,M8344,M8345,M8347,M8348,M8349,M8350,M8351,M8352,M8353,M8354,M8355,M8357,M8358,M8359,M8380,M8381,M8382,M8383,M8384,M8385,M8387,M8388,M8389,M8390,M8391,M8392,M8393,M8394,M8395,M8397,M8398,M8399,M852*,M858*,M859*,M8600,M8608,M8609,M8610,M8618,M8619,M8620,M8628,M8629,M8630,M8638,M8639,M8640,M8648,M8649,M8650,M8658,M8659,M8660,M8668,M8669,M8680,M8688,M8689,M8690,M8698,M8699,M8800,M8801,M8802,M8803,M8804,M8805,M8806,M8807,M8809,M889*,M900*,M901*,M902*,M903*,M904*,M905*,M906*,M907*,M913*,M929,M938,M951,M952,M953,M954,M955,M959,M961,M966,M968,M969,M990*,M991*,M992*,M993*,M994*,M998*,M999*
080011	Acute pyoderma	A46,L00,L01*,L020,L021,L022,L024,L028,L029,L03*,L08*

080020	Herpes zoster	B022,B023,B027,B028,B029
080030	Herpes (excluding herpes zoster), similar disease	B000,B018,B019
080040	Acute viral exanthema	B058,B059,B068,B069,B083
080050	Atopic dermatitis	L20*
080060	Eczema (excluding atopic dermatitis), dermatitis, varicose syndrome without ulcer	L21*,L23*,L24*,L25*,L30*
080070	Chronic pyoderma	L023,L05*
080080	Prurigo, urticaria	L28*,L29*,L50*
080090	Erythema	L510,L518,L519,L52,L53*,L54*,L590
080100	Drug eruption, toxic exanthema	L27*,L511,L512
080110	Bullous disorder	L10*,L11*,L12*,L13*,L14,Q81*
080120	Erythroderma	L26
080130	Keratosis, keratoderma	L82,L83,L850,L851,L853,Q80*,Q828
080140	Inflammatory keratosis	L400,L401,L402,L403,L404,L408,L409,L41*,L42,L43*,L44*
080150	Nail disease	L600,L601,L602,L603,L604,L605,L608,L609,L62*
080160	Atrophic skin disorder	L66*,L900,L901,L902,L903,L904,L906,L908,L909
080170	Lymphoedema after mastectomy	I972
080180	Nevus, phacomatosis	D220,D222,D223,D224,D229,I781,Q825,Q851,Q858,Q859
080190	Alopecia	L63*,L64*,L65*
080200	Hypertrichosis	L68*
080210	Acne, skin disorder (NEC)	L70*,L73*,L980
080220	Disorder of eccrine glands, disorder of apocrine glands	L74*,L75*
080230	Dyschromatosis	L80,L81*
080240	Hyperhidrosis	R61*
080260	Other skin diseases	L22,L45,L67*,L71*,L87*,L944,L945,L946,L948,L949,L981,L985,L986,L989,L99*
090010	Malignant breast tumour	C445,C50*,D05*
090020	Benign breast tumour	D24,D486,N60*,N63

090030	Inflammatory disorder of breast	N61,O91*
090040	Mastoplasia	N62
090050	Breast disorder (NEC)	N64*,O920,O921,Q83*
100010	Polyendocrinopathy	D358,D448
100020	Malignant thyroid tumour	C73,D093,D440,E070
100030	Tumour of endocrine gland and associated tissue	C752,C753,C754,C755,C758,C759,D353,D354,D355,D357,D359,D442,D444,D445,D446
100040	Diabetic ketoacidosis, nonketotic coma	E100,E101,E110,E111,E130,E131,E140,E141
100050	Hypoglycaemia (associated with diabetes treatment)	E160
100060	Type 1 diabetes (excluding diabetic ketoacidosis)	E102,E103,E104,E105,E106,E107,E108,E109
100070	Type 2 diabetes (excluding diabetic ketoacidosis)	E112,E113,E114,E115,E116,E117,E118,E119
100080	Secondary diabetes (excluding diabetic ketoacidosis)	E122,E123,E124,E125,E126,E127,E128,E129,E132,E133,E134,E135,E136,E137,E138,E139
100100	Diabetic foot	L97,M142
100110	Borderline diabetes, impaired glucose tolerance	R730
100120	Obesity	E66*
100130	Benign thyroid nodule	D34,E01*,E04*,E071
100140	Hyperthyroidism	E05*,E061
100150	Chronic thyroiditis	E062,E063,E064,E065,E069,O905
100160	Hypothyroidism	E02,E03*,E078,E890
100170	Acute thyroiditis	E060
100180	Hyperadrenocorticism, adrenocortical tumour	C740,C749,C797,D350,D441,E242,E243,E244,E248,E249,E258,E259,E260,E261,E269,E270
100190	Pheochromocytoma, paraganglioma	C741,D356,D447,E275
100201	Addisonian crisis	E272
100202	Hypoadrenocorticism (NEC)	E250,E271,E273,E274,E896
100210	Hypoglycaemia	E15,E161,E162
100220	Primary hyperparathyroidism, parathyroid tumour	C750,D351,E210,E212,E213,E214,E215
100230	Secondary hyperparathyroidism	E211

100240	Hypoparathyroidism	E20*,E892
100250	Hypopituitarism	E230,E231,E236,E237
100260	Hyperpituitarism	C751,D352,D443,E220,E221,E228,E229,E240,E241
100270	Diencephalohypophysial disease (NEC)	E233,F500,F502
100280	Diabetes insipidus	E232
100285	Syndrome of inappropriate ADH secretion	E222
100290	Glucose control/pancreatic secretion disorder, other endocrine diseases	E00*,E079,E163,E168,E169,E278,E279,E28*,E29*,E30*,E31*,E340,E341,E342,E344,E345,E348,E349,E35*,E891,E893,E894,E895,E898,E899,Q96*,Q980,Q981,Q982,Q983,Q984
100300	Metabolic disorder other than diabetes mellitus	E740,E78*,E790,E80*,E830,E831,E832,E833,E834,E838,E839
100310	Renovascular hypertension	I150,I701,I722
100320	Endocrine hypertension (NEC)	I151,I152,I158,I159
100330	Nutritional deficiency (NEC)	E40,E41,E42,E43,E44*,E45,E46,E50*,E511,E518,E519,E52,E53*,E54,E55*,E56*,E58,E59,E60,E61*,E63*,E64*,E65,E67*,E68
100335	Metabolic disorder (NEC)	E70*,E71*,E72*,E73*,E741,E742,E743,E744,E748,E749,E75*,E76*,E77*,E791,E798,E799,E84*,E88*,E90
100340	Diabetes (NEC)	E120,E121,E142,E143,E144,E145,E146,E147,E148,E149
100360	Dwarfism	E343
100370	Amyloidosis	E85*
100380	Dehydration	E86
100391	Hypokalaemia	E268,E876
100392	Disorderof calcium metabolism	E835
100393	Other humoral/electrolyte/acid-base balance disorders	E870,E871,E872,E873,E874,E875,E877,E878
110010	Malignant renal tumour	C64,C790
110020	Malignant genital tumour (NEC)	C638,C639,C688,C689
110030	Benign renal tumour	D300,D410

110040	Benign genital tumour (NEC)	D076,D290,D291,D297,D299,D307,D309,D40*,D411,D412,D413,D414,D417,D419
110050	Retroperitoneal disease	C786,D200,D483
110060	Malignant tumour of renal pelvis/ ureter	C65,C66,D091
110070	Urinary bladder tumour	C67*,C680,C681,C791,D090,D303
110080	Malignant prostatic tumour	C61,C637,D075
110090	Malignant penile tumour	C60*,D074
110100	Testicular tumour	C62*,C630,C631,C632,D176,D292,D293
110110	Other upper urinary tract diseases	D301,D302,N281,N288
110120	Upper urinary lithiasis	N20*
110130	Lower urinary lithiasis	N21*
110140	Vesicoureteral reflux	N137,N138,N139,Q627
110150	Neurogenic bladder	G834,N31*
110160	Inflammation of lower urinary tract	A562,N30*,N34*
110170	Urethral stenosis	N35*,N991
110180	Urethral tumour	D304,N363
110190	Urinary incontinence	N393,N394
110200	Benign prostatic hyperplasia	N40
110220	Inflammatory disease of male genital organs	N41*,N45*
110230	Disease of penis/prepuce	N47,N48*
110240	Intrascrotal disease (excluding malignant and inflammatory disease)	D294,I861,N43*,N44
110250	Disorder of male genital organs	N46,N508
110260	Nephrotic syndrome	N04*
110270	Acute nephritic syndrome/rapidly progressive nephritic syndrome	N000,N001,N005,N008,N01*
110280	Chronic nephritic syndrome/chronic interstitial nephritis/chronic renal failure	I120,I129,N02*,N03*,N05*,N06*,N07*,N08*,N11*,N12,N14*,N18*,N391,N392
110290	Acute renal failure	K767,N17*
110310	Renal infection	N10,N151

110320	Renal and urinary system disease (NEC)	I823,N150,N158,N159,N16*,N19,N22*,N23,N25*,N26,N27*,N289,N29*,N32*,N33*,N360,N361,N362,N368,N369,N37*,N390,N398,N399,N42*,N49*,N500,N501,N509,N51*,N990,N992,N993,N994,N995,N998,N999,Q624,Q628,T19*
110420	Hydronephrosis (NEC)	N130,N131,N132,N133,N134,N135,N136
110430	Renal artery embolism	N280
120010	Malignant tumour of ovary/uterine appendages	C56,C57*,C796
120020	Malignant tumour of cervix/corpus of uterus	C53*,C54*,C55,D06*,D070,D073,N87*
120030	Malignant vulval tumour	C51*,D071,N900,N901,N902,N903,N904
120040	Malignant vaginal tumour	C52,D072,N890,N891,N892,N893,N894
120050	Malignant tumour of placenta, hydatidiform mole	C58,D392,O01*
120060	Benign uterine tumour	D25*,D26*,D390
120070	Benign ovarian tumour	D27,D391
120080	Benign tumour of female genital organs (NEC)	D28*,D397,D399
120090	Prolapse of genital organs	N81*
120100	Endometriosis	N800,N801,N802,N803,N804,N805,N806,N808,N809
120110	Inflammatory disease of uterus/adnexa	N70*,N71*,N72,N73*,N74*
120120	Noninflammatory disease of ovary/fallopian tube/broad ligament	N83*
120130	Ectopic pregnancy	O00*
120140	Miscarriage	O02*,O03*,O04*,O05*,O06*,O07*,O08*
120150	Haemorrhage in early pregnancy	O20*
120160	Hypertension or other diseases associated with pregnancy/labour/puerperium	O10*,O11,O12*,O13,O14*,O15*,O16,O21*,O22*,O23*,O25,O26*
120170	Premature labour, threatened premature labour	O470,O471,O479,O60

120180	Maternal care related to the fetus and amniotic cavity and possible delivery problems	O30*,O31*,O32*,O33*,O34*,O35*,O36*,O40,O41*,O42*,O43*,O44*,O45*,O46*,O48
120190	Inflammatory disease of female genital organs (NEC)	N75*,N76*,N77*
120200	Diabetes during pregnancy	O24*
120210	Fistula including female genital organs	N82*
120220	Polyp of female genitalia	N84*
120230	Noninflammatory disorder of uterus	N85*,N86,N88*
120240	Noninflammatory disorder of vagina and vulva	N895,N896,N897,N898,N899,N905,N906,N907,N908,N909
120250	Clinical condition associated with reproduction/menstrual cycle	N91*,N92*,N93*,N94*,N95*,N96,N97*
120260	Dystocia with complication of labour or obstetric operation	O61*,O62*,O63*,O64*,O65*,O66*,O67*,O68*,O69*,O70*,O71*,O72*,O73*,O74*,O75*,O81*,O82*,O83*,O84*
120270	Clinical condition associated with pregnancy, labour or puerperium	O28*,O29*,O85,O86*,O87*,O88*,O89*,O900,O901,O902,O903,O904,O908,O909,O922,O923,O924,O925,O926,O927,O95,O96,O97,O98*,O99*
120280	Pelvic varix, external pudendal varix	I862,I863
120300	Complication associated with artificial insemination	N98*
130010	Acute leukaemia	C910,C912,C913,C917,C919,C920,C922,C923,C924,C925,C927,C929,C930,C932,C937,C939,C940,C942,C943,C944,C945,C947,C950,C952,C957,C959
130020	Hodgkin disease	C810,C811,C812,C813,C817,C819
130030	Non-Hodgkin lymphoma	C820,C821,C822,C827,C829,C830,C831,C832,C833,C834,C835,C836,C837,C838,C839,C840,C841,C842,C843,C844,C845,C850,C851,C857,C859
130040	Multiple myeloma, malignant neoplasm of immune system	C880,C881,C882,C883,C887,C889,C900,C901,C902
130050	Chronic leukaemia, myeloproliferative disease	C911,C914,C915,C921,C931,C951,D45,D471,D473

130060	Osteomyelodysplasia	D460,D461,D462,D463,D464,D467,D469
130070	Leucocytic disease (NEC)	D70,D720,D721,D728,D729
130080	Aplastic anaemia	D600,D601,D609,D610,D611,D612,D613,D619
130090	Anaemia (NEC)	D500,D501,D508,D509,D510,D511,D512,D513,D518,D519,D520,D521,D528,D529,D530,D531,D532,D538,D539,D550,D551,D552,D559,D560,D561,D562,D563,D564,D569,D570,D571,D572,D573,D580,D581,D582,D588,D589,D590,D591,D592,D593,D594,D595,D596,D599,D62,D640,D641,D642,D643,D644,D648,D649
130100	Disseminated intravascular coagulation	D65,D683
130110	Haemorrhagic disease (NEC)	D691,D692,D693,D694,D695,D696,D698,D699
130111	Allergic purpura	D690
130120	Haematological disease (NEC)	C941,D553,D558,D568,D578,D598,D608,D618,D63*,D73*,D74*,D75*,D76*,D77,D89*
130130	Coagulopathy (NEC)	D680,D681,D682,D684,D688,D689
130140	Haematopoietic organ disease (NEC)	D470,D472,D477,D479
130150	Primary immunodeficiency syndrome	D71,D80*,D81*,D82*,D83*,D84*
140010	Disorder associated with shortened gestation period or low birth weight	P07*
140020	Birth asphyxia	P21*
140030	Neonatal disorder with onset during the perinatal period	P00*,P01*,P02*,P03*,P04*,P05*,P08*,P10*,P11*,P12*,P13*,P15*,P20*,P22*,P23*,P24*,P25*,P26*,P28*,P29*,P35*,P36*,P37*,P38,P39*,P50*,P51*,P52*,P53,P54*,P55*,P56*,P57*,P58*,P598,P60,P61*,P70*,P71*,P72*,P74*,P75,P76*,P77,P780,P781,P782,P783,P789,P80*,P81*,P83*,P90,P91*,P92*,P93,P94*,P95,P96*
140040	Neonatal jaundice	P590,P591,P592,P593,P599
140050	Chronic respiratory disease with onset during perinatal period	P27*
140060	Neonatal gastric rupture	P788
140070	Malformation of cranial or facial bones	Q670,Q671,Q672,Q673,Q75*,Q870

140080	Malformation of brain or spine	Q00*,Q01*,Q02,Q03*,Q04*,Q05*,Q06*,Q07*,Q760
140090	Congenital nasolacrimal duct obstruction	Q105
140100	Congenital ocular anomaly	Q101,Q102,Q103,Q104,Q106,Q107,Q11*,Q14*,Q158,Q159
140110	Nasal anomaly (congenital/acquired)	M950,Q301,Q302,Q303,Q308,Q309
140120	Ankyloglossia	Q381
140130	Macroglossia	Q382
140140	Congenital disorder of palate/lips	Q35*,Q36*,Q37*
140150	Laryngeal web, congenital laryngeal stenosis	Q310,Q318
140160	Choanal atresia	Q300
140170	Median cervical cyst/lateral cervical cyst	Q188,Q892
140190	Microtia/auricular abnormality	Q160,Q171,Q172,Q173,Q174,Q175
140200	Ear atresia	Q161
140210	Preauricular sinus or accessory auricle	Q170,Q181
140220	Ear disease (NEC)	Q162,Q164,Q165,Q169,Q178,Q179,Q180,Q182,Q183,Q184,Q185,Q186,Q187,Q189
140230	Laryngeal disease (NEC)	Q311,Q312,Q313,Q314,Q319
140240	Congenital anomaly of oral cavity	Q380,Q383,Q384,Q385,Q386,Q387
140250	Congenital pharyngeal anomaly	Q388
140260	Deformation and congenital anomaly of thoracic cage	Q676,Q677,Q678,Q765,Q766,Q767
140270	Congenital pulmonary anomaly	Q330,Q331,Q332,Q333,Q334,Q335,Q336,Q338,Q339,Q340
140280	Congenital anomaly of respiratory tract	Q32*,Q348,Q349
140290	Patent ductus arteriosus	Q250
140300	Atrial septal defect	Q211
140310	Ventricular septal defect	Q210
140320	Atrioventricular septal defect	Q212

140330	Other cardiac anomalies with left-to-right shunt	Q214,Q245,Q254,Q257,Q263
140340	Cardiac anomaly without shunt	Q231,Q232,Q233,Q253,Q256
140350	Tetralogy of Fallot	Q213
140360	Congenital cardiac anomaly in neonate and infant	Q200,Q201,Q202,Q203,Q220,Q226,Q234,Q251, Q252,Q255,Q262
140370	Other complex cardiac anomalies	Q204,Q205,Q224
140380	Other congenital cardiovascular anomalies	Q208,Q209,Q218,Q219,Q221,Q222,Q223,Q225, Q228,Q229,Q230,Q238,Q239,Q240,Q241,Q242, Q243,Q244,Q246,Q248,Q249,Q258,Q259,Q260, Q261,Q264,Q265,Q266,Q268,Q269
140390	Congenital oesophageal anomaly	Q39*
140400	Congenital gastric/upper digestive tract anomaly	Q402,Q403,Q408,Q409,Q458,Q459
140410	Congenital hypertrophic pyloric stenosis	Q400
140420	Small-intestinal atresia	Q41*
140430	Congenital intestinal anomaly	Q43*
140440	Anal atresia	Q42*
140450	Congenital biliary tract anomaly (ectasis)	Q444,Q445,Q453
140460	Congenital biliary tract anomaly (atresia)	Q442,Q443
140470	Congenital hepatobiliary and pancreatic system anomaly	Q440,Q441,Q446,Q447,Q450,Q451,Q452
140480	Congenital abdominal wall abnormality	Q792,Q793
140490	Congenital disorder of hand and foot	Q66*,Q681,Q682,Q684,Q685,Q688,Q710,Q711, Q712,Q713,Q714,Q715,Q718,Q719,Q720,Q721, Q722,Q723,Q724,Q725,Q726,Q727,Q729,Q73*, Q740,Q749
140500	Congenital osteochondral dysplasia	Q77*,Q78*,Q796,Q798
140510	Congenital hip disease	Q65*
140520	Congenital femoral disease	Q683
140530	Congenital anomaly of toe	Q69*,Q70*,Q716

140550	Congenital cystic kidney disease	Q61*
140560	Congenital hydronephrosis	Q620,Q621,Q622,Q623
140570	Congenital upper urinary tract disease	Q625,Q626,Q63*
140580	Congenital lower urinary tract disease	Q54*,Q55*,Q64*
140590	Cryptorchism	Q53*
140600	Congenital anomaly of female genital organs	Q51*,Q52*
140620	Other congenital anomalies	Q206,Q270,Q271,Q272,Q273,Q274,Q278,Q279,Q288,Q289,Q50*,Q56*,Q60*,Q674,Q728,Q741,Q742,Q743,Q748,Q768,Q769,Q794,Q795,Q799,Q820,Q821,Q822,Q823,Q824,Q829,Q84*,Q86*,Q871,Q872,Q873,Q874,Q875,Q878,Q890,Q891,Q893,Q894,Q897,Q898,Q899
150010	Viral enteritis	A08*,A09
150020	Bacterial enteritis	A00*,A01*,A020,A021,A022,A029,A030,A031,A032,A033,A039,A050,A052,A053,A054,A058,A059
150030	Viral meningitis	A87*
150040	Febrile convulsion	R560
150050	Acute encephalitis/encephalopathy	A830,A831,A832,A833,A834,A835,A836,A84*,A850,A851,A852,A86,G934
150060	Acute nephritis	N002,N003,N004,N006,N007,N009
150070	Kawasaki disease	M303
150100	Maltreatment syndrome	T74*
150110	Chromosome aberration (excluding Turner and Klinefelter syndromes)	Q90*,Q91*,Q92*,Q93*,Q95*,Q97*,Q985,Q986,Q987,Q988,Q989,Q99*
160010	Other malignant tumours	C457,C459,C761,C762,C767,C768,C778,C779,C788,C794,C798,C80,C97,D019,D040,D043,D099,D165
160030	Other neoplasms	D139,D157,D159,D164,D175,D177,D181,D213,D215,D230,D232,D233,D234,D239,D367,D369,D379,D449,D480,D482,D484,D485,D487,D489
160040	Haemangioma, lymphangioma	D180

160060	Mental/behavioural disorder	F00*,F02*,F03,F04,F05*,F06*,F07*,F09,F100,F101,F103,F104,F105,F106,F108,F109,F11*,F12*,F13*,F14*,F15*,F16*,F17*,F18*,F19*,F20*,F21,F22*,F23*,F24,F25*,F28,F29,F30*,F31*,F32*,F33*,F34*,F38*,F39,F40*,F41*,F42*,F43*,F44*,F45*,F48*,F501,F503,F504,F505,F508,F509,F51*,F52*,F53*,F54,F55,F59,F60*,F61,F62*,F63*,F64*,F65*,F66*,F68*,F69,F70*,F71*,F72*,F73*,F78*,F79*,F80*,F81*,F82,F83,F84*,F88,F89,F90*,F91*,F92*,F93*,F94*,F95*,F98*,F99
160160	Sepsis and other infectious diseases	A028,A038,A060,A061,A062,A063,A066,A067,A068,A069,A07*,A15*,A180,A181,A184,A185,A186,A187,A188,A20*,A21*,A22*,A23*,A24*,A25*,A26*,A27*,A28*,A30*,A311,A318,A32*,A33,A34,A35,A36*,A37*,A38,A39*,A40*,A41*,A42*,A43*,A44*,A482,A484,A488,A49*,A50*,A51*,A52*,A53*,A54*,A55,A560,A561,A563,A564,A568,A57,A58,A59*,A60*,A638,A64,A65,A66*,A67*,A68*,A690,A692,A698,A699,A70,A71*,A74*,A75*,A77*,A78,A79*,A80*,A838,A839,A90,A91,A92*,A93*,A94,A95*,A96*,A98*,A99,B001,B002,B003,B004,B005,B007,B008,B009,B03,B04,B07,B080,B081,B082,B084,B085,B088,B09,B25*,B260,B261,B262,B268,B269,B30*,B33*,B34*,B35*,B36*,B370,B372,B373,B374,B375,B376,B377,B378,B379,B38*,B39*,B40*,B41*,B42*,B43*,B442,B447,B448,B449,B46*,B47*,B48*,B49,B50*,B51*,B52*,B53*,B54,B55*,B56*,B57*,B58*,B60*,B64,B650,B651,B652,B653,B658,B660,B661,B662,B663,B665,B668,B669,B67*,B68*,B69*,B70*,B71*,B72,B73,B74*,B75,B76*,B77*,B78*,B79,B80,B81*,B82*,B83*,B85*,B86,B87*,B88*,B89,B90*,B91,B92,B94*,B95*,B96*,B97*,B99,T793

160220	Other abnormal findings	H92*,R00*,R01*,R02,R03*,R041,R05,R06*,R07*,R09*,R10*,R11,R12,R13,R14,R15,R16*,R17,R18,R19*,R20*,R21,R220,R221,R222,R223,R224,R227,R229,R23*,R25*,R26*,R27*,R29*,R30*,R31,R32,R33,R34,R35,R36,R39*,R40*,R41*,R42,R43*,R44*,R45*,R46*,R47*,R48*,R49*,R50*,R51,R52*,R53,R54,R55,R568,R57*,R58,R59*,R60*,R62*,R63*,R64,R68*,R69,R70*,R71,R72,R739,R74*,R75,R76*,R77*,R78*,R79*,R80,R81,R82*,R83*,R84*,R85*,R86*,R87*,R89*,R90*,R91,R92,R930,R931,R932,R933,R934,R935,R936,R937,R938,R940,R941,R942,R943,R944,R945,R946,R947,R948,R95,R96*,R98,R99
160230	Phrenic nerve palsy due to birth injury	P142
160260	Acquired immunodeficiency syndrome	B20*,B21*,B22*,B23*,B24
160270	HIV related disease	C46*
160280	Haemophilia	D66,D67
160300	Intraorbital foreign body	H05*,S054
160310	Traumatic/secondary ocular disorder (intraocular injury)	H261,H335,H403,H444,S051
160320	Intraocular foreign body, ocular penetrating wound	H446,H447,S055,S056,T150,T151,T158,T159
160330	Eyelid laceration	S001,S002,S011
160340	Laceration of sclera/cornea, rupture of eyeball	S052,S053,S057,S058,S059
160350	Ocular/orbital injury	S050
160360	Ocular heat injury or chemical burn	T26*
160370	Oral/digestive tract foreign body	T180,T188
160380	Injury or laceration of tongue	S015
160390	Pharyngeal injury	S100,S101,S110
160400	Nasal bone fracture	S022*
160410	Facial injury	S003,S004,S005,S012,S013,S014,S025*

160420	Craniofacial injury	S000,S007,S008,S009,S010,S017,S018,S019,S020*,S021*,S023*,S024*,S026*,S027*,S028*,S029*,S06*,S07*,S08*,S09*
160440	Foreign body in ear	T16
160450	Laryngeal/tracheal foreign body	T17*
160460	Injury of thoracic parenchymal organ	S243,S244,S246,S254,S255,S257,S258,S259,S27*,S28*
160470	Thoracic bony injury	S222*,S223*,S224*,S225*,S228*,S229*,S234,S235,S29*
160480	Injury of thoracic great vessel	S250,S251,S252,S253
160490	Cardiac trauma	S26*
160500	Gastric trauma	S363*
160510	Hepatic trauma	S361*
160520	Pancreatic trauma	S362*
160530	Splenic trauma	S360*
160540	Intestinal tract trauma (from duodenum to rectum)	S364*,S365*,S366*,S367*,S368*,S369*
160550	Abdominal vessel injury	S35*
160560	Oesophageal/gastric foreign body	T181,T182,T183,T184
160570	Anal foreign body	T185
160580	Upper-limb open wound	S20*,S21*,S30*,S310,S311,S411,S618,S619,T111
160590	Nerve injury of extremity	S44*,S54*,S64*,S74*,S84*,S94*,T113
160600	Vascular injury of extremity	S45*,S55*,S65*,S75*,S85*,S95*,T114
160610	Muscle tendon injury of extremity	M620*,M621*,M623*,M626*,M628*,M629*,M660*,M661*,M662*,M663*,M664*,M665*,S46*,S534,S56*,S633,S634,S635,S636,S637,S66*,S731,S76*,S836,S86*,S934,S935,S936,S96*,T112,T115
160620	Elbow/knee trauma (including sports disorder and other conditions)	M23*,M704*,M705*,M8306,M8316,M8326,M8336,M8346,M8356,M8386,M8396,M932,S532,S533,S830,S831,S832,S833,S834,S835,S837
160630	Periarticular fracture of ankle joint	M8436,S8220,S8230,S8240,S8290
160640	Traumatic amputation	S48*,S58*,S68*,S78*,S88*,S98*,T05*,T096,T116,T136,T147

160650	Compartment syndrome	M622*,M6240,M6242,M6243,M6247,M6248,M6249,M625*,T796
160660	Subcutaneous injury of soft tissue/ crush injury, open wound	S40*,S410,S417,S418,S47,S50*,S51*,S57*,S600,S601,S602,S607,S608,S609,S610,S611,S617,S670,S70*,S71*,S77*,S80*,S81*,S87*,S90*,S91*,S97*
160670	Multiple injuries (shoulder, arm or leg)	S49*,S59*,S69*,S79*,S89*,S99*
160680	Soft tissue disorder of shoulder joint	S434,S435,S436,S437
160690	Impairment from fracture of thoracic or lumbar vertebra or lower (including thoracic/lumbar spinal cord injury)	S220*,S221*,S230,S231,S232,S233,S240,S241,S242,S245,S320*,S330,S331,S332,S333,S340,S341,S342,S343,S344,S345,T08*
160700	Fracture of clavicle or scapula	S4200,S4210
160710	Compound fracture of clavicle or scapula	S4201,S4211
160720	Periarticular fracture/dislocation of shoulder	M2431,M2441,S4220,S4230,S4270,S4280,S4290,S430,S431,S432,S433
160730	Periarticular compound fracture of shoulder	S4221,S4231,S4271,S4281,S4291
160740	Periarticular fracture/dislocation of elbow	S4240,S5200,S5210,S530,S531
160750	Periarticular compound fracture of elbow	S4241,S5201,S5211
160760	Fracture of forearm	S5220,S5230,S5240,S5250,S5270,S5280,S5290
160770	Compound fracture of forearm	S5221,S5231,S5241,S5251,S5271,S5281,S5291
160780	Periarticular fracture/dislocation of wrist	S5260,S6200,S6210,S6220,S6230,S6240,S6250,S6260,S6270,S6280,S630,S631,S632
160790	Compound periarticular fracture of wrist	S5261,S6201,S6211,S6221,S6231,S6241,S6251,S6261,S6271,S6281
160800	Fracture of proximal femur	M2435,M2445,S7200,S7210,S7220,S7230,S7270,S7280,S7290,S730
160810	Compound fracture of proximal femur	S7201,S7211,S7221,S7231,S7271,S7281,S7291
160820	Periarticular fracture/dislocation of knee	S7240,S8200,S8210,S8270

160830	Compound periarticular fracture of knee	S7241,S8201,S8211,S8271
160840	Compound periarticular fracture of leg or ankle joint	S8221,S8231,S8241,S8291
160850	Fracture/dislocation of ankle joint or foot	M8437,S8250,S8260,S8280,S9200,S9210,S9220, S9230,S9240,S9250,S9270,S930,S931,S932,S933
160860	Fracture, dislocation or compound fracture of ankle joint/foot	S8251,S8261,S8281,S9201,S9211,S9221,S9231,S9241,S9251,S9271
160870	Cervical spine and cord injury	S120*,S121*,S122*,S127*,S129*,S130,S131,S132,S133,S134,S136,S140,S141,S142,S143,S144,S145,S146,S346
160880	Upper limb/forearm injury (NEC)	T118
160890	Deformity due to malunion or nonunion (excluding upper limb)	M8405,M8406,M8407,M8415,M8416,M8417,M8425,M8426,M8427,M8445,M8446,M8447,M8485,M8486,M8487,M960,T911,T912,T931,T932
160895	Deformity due to malunion or nonunion (upper limb)	M8401,M8402,M8403,M8404,M8411,M8412,M8413,M8414,M8421,M8422,M8423,M8424,M8431,M8432,M8433,M8434,M8441,M8442,M8443, M8444,M8491,M8492,M8493,M8494,T921,T922
160950	Renal/ureteral trauma	S370*,S371*
160960	Vesical/urethral trauma	S372*,S373*
160970	Male genital organ trauma	S380,S382
160980	Pelvic fracture	S321*,S322*,S323*,S324*,S325*,S327*,S328*,S334,S335,S336,S337
160990	Multiple injury	T00*,T01*,T02*,T03*,T04*,T06*,T07
160995	Respiratory tract burn	T27*

161000	Burn/chemical burn/frostbite/ fulminating injury	L55*,L56*,T200,T201,T202,T203,T204,T205,T206,T207,T210,T211,T212,T213,T214,T215,T216,T217,T220,T221,T222,T223,T224,T225,T226,T227,T230,T231,T232,T233,T234,T235,T236,T237,T240,T241,T242,T243,T244,T245,T246,T247,T250,T251,T252,T253,T254,T255,T256,T257,T280,T281,T282,T283,T284,T285,T286,T287,T290,T291,T292,T293,T294,T295,T296,T297,T300,T301,T302,T303,T304,T305,T306,T307,T31*,T32*,T330,T331,T332,T333,T334,T335,T336,T337,T338,T339,T340,T341,T342,T343,T344,T345,T346,T347,T348,T349,T350,T351,T352,T353,T354,T355,T356,T357,T750,T752,T754
161020	Body temperature abnormality	T67*,T68,T69*
161030	Barometric injury	T70*
161040	Sequela or subsequent complication of injury	T90*,T910,T913,T914,T915,T918,T919,T920,T923,T924,T925,T926,T928,T929,T930,T933,T934,T935,T936,T938,T939,T94*,T95*,T96,T97,T98*
161050	Other injuries	S03*,S041,S042,S043,S044,S045,S046,S047,S048,S049,S107,S108,S109,S111,S112,S117,S118,S119,S128*,S135,S15*,S16,S17*,S18,S19*,S312,S313,S314,S315,S317,S318,S348,S374*,S375*,S376*,S377*,S378*,S379*,S381,S383,S39*,S678,S929*
161060	Unspecified injury (NEC)	T090,T091,T092,T093,T094,T095,T098,T099,T10*,T110,T119,T12*,T130,T131,T132,T133,T134,T135,T138,T139,T140,T141,T142*,T143,T144,T145,T146,T148,T149,T189,T288,T289,T66,T71,T73*,T751,T753,T758,T78*,T794,T795,T797,T798,T799,T80*,T81*,T820,T822,T823,T824,T825,T826,T827,T828,T829,T83*,T84*,T85*,T86*,T87*,T880,T881,T882,T883,T884,T885,T886,T888,T889

161070	Drug intoxication (other intoxications)	T36*,T37*,T38*,T39*,T40*,T41*,T42*,T43*,T44*,T45*,T46*,T47*,T48*,T49*,T50*,T51*,T52*,T53*,T54*,T55,T56*,T57*,T58,T59*,T60*,T61*,T62*,T63*,T64,T65*,T887

NEC: Not elsewhere classified (used in ICD-10)

*: Corresponds to arbitrary numerals

"M!!!!" in the ICD-10 column of No. 071030 means "ICD-10 code numbers starting with 'M'NEC".
